# Narrowband UVB phototherapy reduces TNF production by B‐cell subsets stimulated via TLR7 from individuals with early multiple sclerosis

**DOI:** 10.1002/cti2.1197

**Published:** 2020-10-15

**Authors:** Stephanie Trend, Jonatan Leffler, Matthew N Cooper, Scott N Byrne, Allan G Kermode, Martyn A French, Prue H Hart

**Affiliations:** ^1^ Telethon Kids Institute University of Western Australia Perth WA Australia; ^2^ Centre for Neuromuscular and Neurological Disorders Perron Institute for Neurological and Translational Science University of Western Australia Perth WA Australia; ^3^ School of Medical Sciences Faculty of Medicine and Health The University of Sydney Sydney NSW Australia; ^4^ Centre for Immunology and Allergy Research Westmead Institute for Medical Research Westmead NSW Australia; ^5^ Institute for Immunology and Infectious Disease Murdoch University Perth WA Australia; ^6^ UWA Medical School and School of Biomedical Sciences University of Western Australia Perth WA Australia

**Keywords:** B cells, IL‐10, multiple sclerosis, TLR7, TNF, UVB radiation

## Abstract

**Objectives:**

At the end of a 60‐day course of narrowband UVB phototherapy, administered to individuals with early multiple sclerosis, there were changes in the relative proportions of circulating B‐cell subsets. This study investigated phototherapy‐associated changes to cytokine responses of B cells when exposed to a TLR7 ligand.

**Methods:**

PBMCs from participants of the PhoCIS (Phototherapy for Clinically Isolated Syndrome) trial taken before (day 1) and after phototherapy for 8 weeks (day 60) were incubated with, or without, the TLR7 ligand, R848, for 18 h. Production of TNF and IL‐10 in seven B‐cell subsets was examined, with cytokine responses in each individual at day 60, adjusted for responses at day 1. Paired PBMCs were from participants administered phototherapy (*n* = 7) or controls (*n* = 6).

**Results:**

At day 60, significantly fewer B cells, particularly marginal zone‐like B cells (CD27^+^/IgD^+^), from participants administered phototherapy produced TNF in response to TLR7 stimulation. When responses by seven B‐cell subsets were analysed together using multivariate methods, a phototherapy‐specific signature was observed. An increased responsiveness from day 1 to day 60 in IgM‐only memory B cells (CD27^+^/IgD^−^/IgM^+^) after TLR7 stimulation also predicted slower progression from CIS to MS. Phototherapy was without significant effect on B‐cell IL‐10 production.

**Conclusions:**

Reduced TNF responses after TLR7 stimulation in marginal zone‐like B cells from participants administered phototherapy suggested treatment‐associated priming effects that were detected upon subsequent polyclonal B‐cell activation. Changes in responsiveness to TLR7 stimulation also suggested that IgM‐only memory B cells may be important in conversion from CIS to MS.

## Graphical abstract

Phototherapy was tested to prevent or delay individuals with clinically isolated syndrome converting to multiple sclerosis. This study investigated the functional impact of phototherapy on circulating B cells. In addition to modulating the proportion of circulating B‐cell subsets, phototherapy reduced the ability of B cells to respond to TLR7 stimulation with pro‐inflammatory TNF production suggesting that phototherapy may limit polyclonal B‐cell activation.

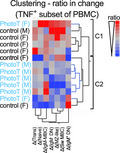

## Introduction

Genetic predisposition to multiple sclerosis (MS) explains only a fraction of the disease risk.^1^ Ecologic studies support the concept of latitude gradients for the incidence, prevalence and mortality of MS, with more disease at higher latitudes where there is generally less sun exposure.^2^, ^3^ Many have suggested that low vitamin D status is responsible for these observations.^4^, ^5^ The main source of 25‐hydroxy vitamin D (25(OH)D) for humans is derived following exposure of skin to the UVB components of sunlight. Additionally, reduced sun exposure and low serum 25(OH)D levels are independent risk factors for the development of multiple sclerosis (MS).^6^ The independence of reduced sun exposure and low 25(OH)D as risk factors for MS suggests that there may be several potentially beneficial effects, other than vitamin D production in UV‐irradiated skin.^7^, ^8^ Furthermore, there have been disappointing results following several trials of vitamin D supplementation to patients with MS, who did not gain the hoped‐for benefit suggested by the inverse associations reported for MS incidence and 25(OH)D levels, as reviewed.^9^, ^10^

To investigate the beneficial effect of UV light on MS further, this group established the PhoCIS trial (Phototherapy for Clinically Isolated Syndrome) whereby participants with the earliest form of MS, namely Clinically Isolated Syndrome (CIS), were given a course of suberythemal narrowband UVB (311–312 nm) phototherapy over 8 weeks (3 sessions/week).^11^ Both those receiving phototherapy and those in the control group (no phototherapy) were followed for 12 months both by brain magnetic resonance imaging (MRI) and by venesection at regular intervals. Many outcomes from the trial have already been published.^11^, ^12^, ^13^, ^14^ When the primary outcome of MRI changes was examined, there was a non‐significant reduction in the rate of converting from CIS to MS within 12 months in participants who received phototherapy. In the control group of 10, all (100%) had converted while only 7 of the 10 participants who received narrowband UVB had progressed and been diagnosed with MS within 12 months.^11^ This group has also investigated UVB‐induced changes in the circulating immune cells of the participants with the aim of illuminating the mechanisms of action of narrowband UVB, and whether they contributed to progression of CIS to MS. The most substantial short‐term changes (over the first 2 months) detected involved B cells,^14^ a cell type whose importance has been highlighted by the successful clinical use of anti‐CD20 monoclonal antibodies to treat MS.^15^ In 2019, we reported that there was a significant decrease in CD27^+^/IgD^−^ memory B cells (MBC) and a significant increase in naive B cells (CD27^−^/IgD^+^) (both as a % of B cells) in participants that received phototherapy compared with those who did not. Recently, following our finding that serum IgG_3_ levels predict time of conversion to MS by CIS participants,^13^ the group reported significant reductions in IgG_3_^+^ B cells with phototherapy.^16^

Of importance, none of these studies examined the function of circulating B cells following phototherapy; previous investigations only examined changes in relative cell numbers.^14^, ^16^ Here, responses to an innate B‐cell stimulus, resiquimod (R848), were investigated via the production of TNF and IL‐10 by B‐cell subsets. R848 is a synthetic ligand of TLR7 and TLR8; however, the latter is not expressed by B cells, enabling detection of TLR7‐specific responses.^17^ TLR7 is an endosomal pattern recognition receptor for ssRNA, and while it is important in responses to viral infections,^18^ it was selected as a stimulation target as it is a well‐established and potent polyclonal B‐cell stimulator. TNF is not only a pro‐inflammatory cytokine but is important as a co‐factor supporting B cell–T cell and B cell–myeloid cell interactions, as reviewed.^19^ IL‐10, in contrast, is the prototypic anti‐inflammatory cytokine and has been shown to be important for identification of human B cells with immune‐regulatory properties.^20^, ^21^

Using multi‐parametric flow cytometry and regression analysis, we were able to show that narrowband UVB phototherapy not only altered the distribution of cells in various B‐cell subsets circulating in participants after 60 days (time of last phototherapy session), but cellular responses *in vitro* to a TLR7 ligand were reduced. In participants administered phototherapy, significantly fewer B cells, particularly marginal zone (MZ)‐like B cells, produced TNF in response to R848, while there were no significant changes detected in the capability of B cells to produce IL‐10 in response to a TLR7 ligand. This study highlights that narrowband UVB phototherapy exerts systemic immunological effects beyond the skin.

## Results

In our original study analysing bloods from the complete PhoCIS cohort, narrowband UVB phototherapy increased the proportion of naive B cells and decreased the proportion of MBC (CD27^+^/IgD^−^) within the B‐cell population.^14^ To further explore the mechanisms and clinical outcomes from this intervention, short‐term cultures on biobanked PBMC samples from a subset of the original participants were performed (Table 1; *n* = 7 Phototherapy, 6 Controls). As there was a significant difference in male–female distribution between the two groups, a secondary analysis adjusting for sex was applied to significant findings from the primary analysis.

**Table 1:** Participant data

	PhotoT (*n* = 7)	Control (*n* = 6)	*P*
Age, years (mean, range)	37.4 (27.3–45.9)	37.3 (28.8–54.3)	0.99
Female sex (*n*, %)	2 (28.6%)	5 (83.3%)	0.04*
Days since MRI diagnosis at enrolment (median, range)	32 (26–94)	22 (0–91)	0.50
Days until MRI relapse from enrolment (median, range)	62 (61–339)	138 (45–357)	0.65

**Figure 1:**
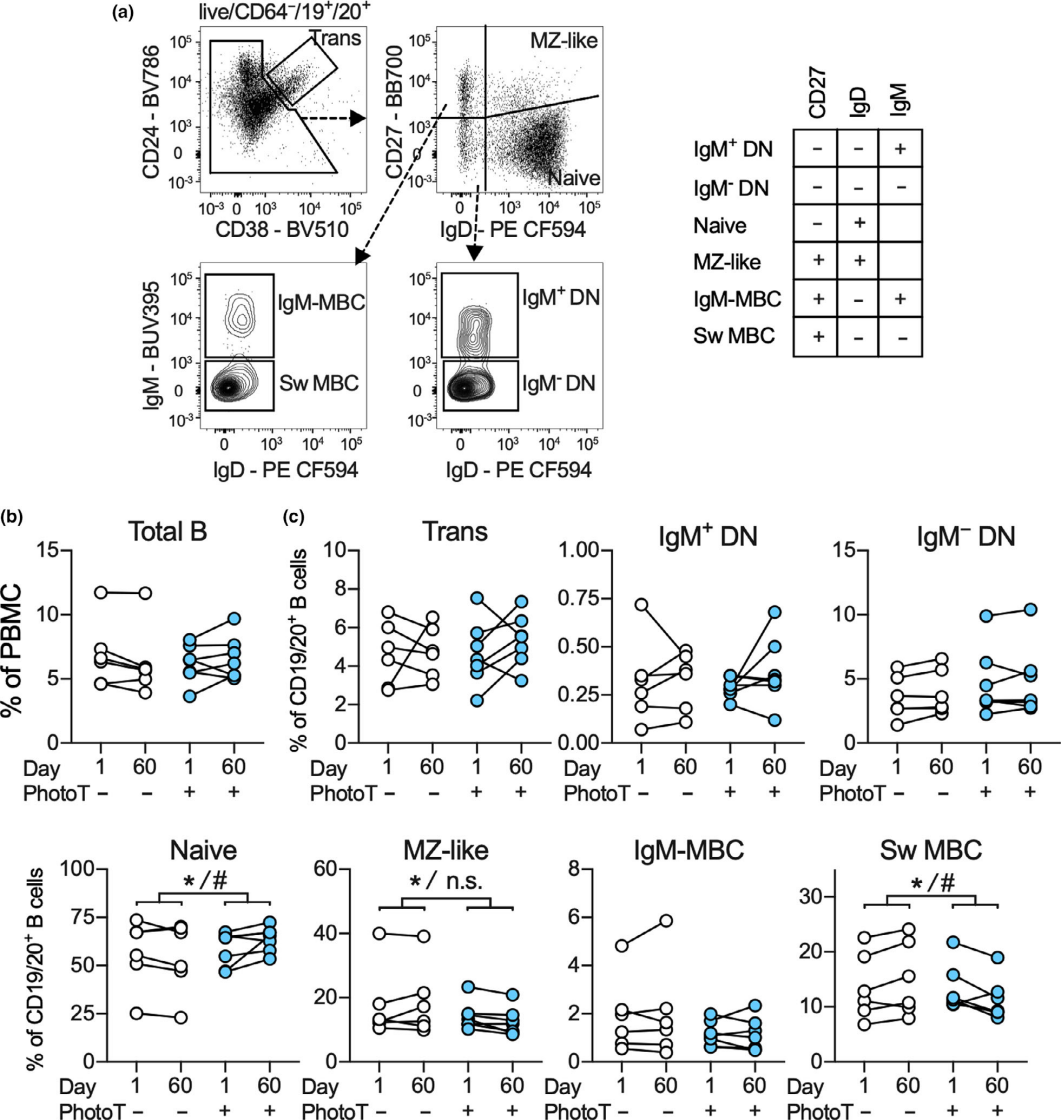
**(a)** Gating strategy and B‐cell subset definitions utilised throughout the study. **(b, c)** Abundance of total B cells **(b)** and proportion of B‐cell subsets within the B‐cell population **(c)** in the control (−PhotoT) and phototherapy (+PhotoT) groups at day 1 and 60. Data in **a** are representative from one individual. Data in **b, c** are displayed as individual data points for each participant (*n*_control_ = 6, *n*_PhotoT_ = 7) measured once. Significance of impact of phototherapy was calculated using multiple regression analysis for abundance at day 60 adjusted for phototherapy and abundance at day 1 and is indicated by *. For significant findings, a secondary analysis was employed to also adjust for the sex of each participant and is indicated by ^#^ according to, *^/#^, *P* < 0.05, IgM‐MBC, IgM‐only MBC.

For this study, B‐cell subsets were identified in the CD19^+^/CD20^+^ population as per Figure 1a and included IgM^+^ and IgM^−^ double‐negative (DN), naive and MZ‐like B cells, IgM‐only MBC and switched MBC (Sw MBC). Using paired‐multiple regression analysis and adjusting for participant‐specific values at baseline, an impact of phototherapy on abundance of total B cells in day 60 samples was not detected (Figure 1b). However, we again observed that samples from participants who underwent phototherapy had a significantly higher proportion of naive B cells and also demonstrated lower proportions of MZ‐like B cells and Sw MBC after 60 days compared to controls (Figure 1c), which were not assessed previously.^14^ After adjusting for sex in the secondary analysis, the effect of phototherapy on naive and Sw MBC but not MZ‐like cells was significant (Figure 1c).

**Figure 2:**
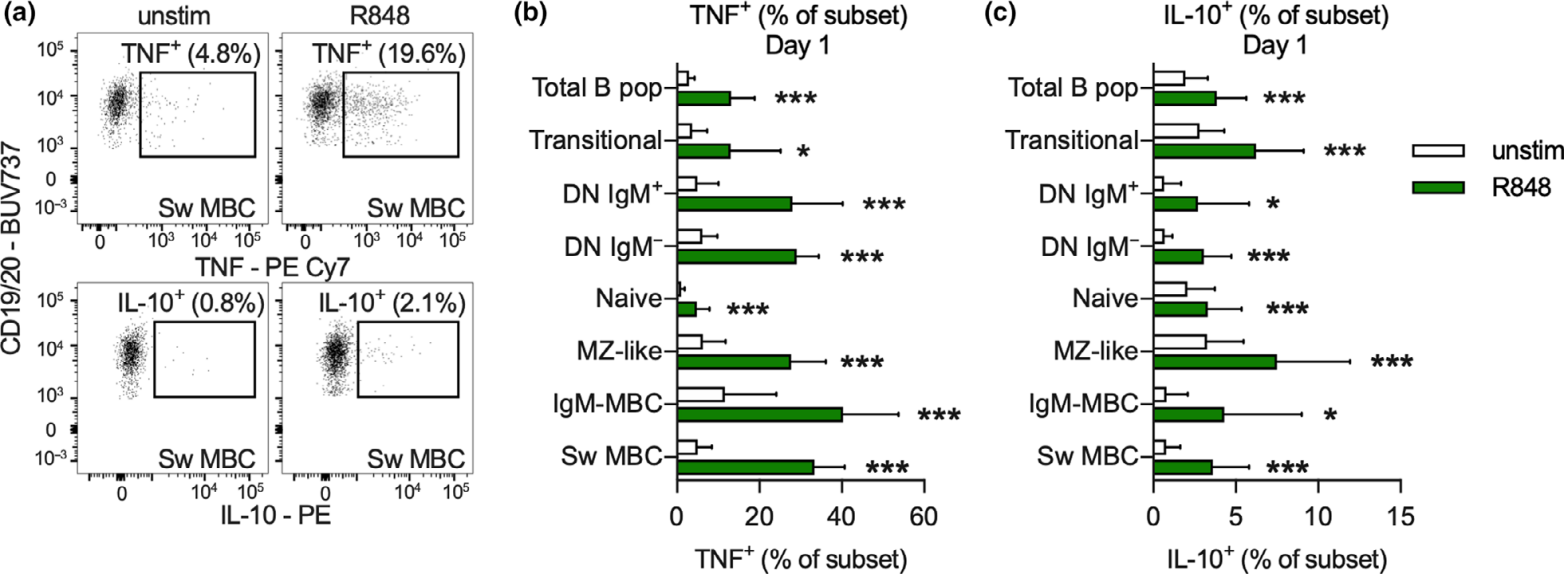
**(a)** Gating for TNF^+^ and IL‐10^+^ Sw MBC in unstimulated (unstim) and R848‐stimulated PBMC. **(b, c)** Proportion of TNF^+^ **(b)** and IL‐10^+^ **(c)** across each B‐cell subset in unstimulated and R848‐stimulated cultures of PBMCs collected at day 1 of the trial. Data in **a** are representative from one participant, and in **b, c**, data are displayed as the mean ± SD in each cell subset and culture condition from *n* = 13 participants measured once. Significance of difference following stimulation was calculated using the Student’s *t*‐test and indicated as *P < 0.05, ***P < 0.001. IgM‐MBC, IgM‐only MBC; n.s., not significant.

Using short‐term (18 h) PBMC cultures, the ability of B‐cell subsets to respond to R848 was assessed by quantifying subsequent production of TNF and IL‐10 using flow cytometry as exemplified for Sw MBC (Figure 2a). When PBMCs from samples collected prior to phototherapy (day 1) were stimulated with R848, the percentage of TNF^+^ (Figure 2b) and IL‐10^+^ (Figure 2c) cells within each B‐cell subset increased from that measured in unstimulated cultures. A large number of IgM‐only MBC (mean 40.3%) and Sw MBC (mean 33.4%) produced TNF while a high proportion of MZ‐like B cells (mean 7.5%) and transitional B cells (mean 6.2%) produced IL‐10. Culture and stimulation also induced minor changes in the abundance of total B cells (as a % of PBMC) as well as several minor changes in B‐cell subset frequency distribution, none of which impacted subsequent analysis (Supplementary figure 1a, b).

**Figure 3:**
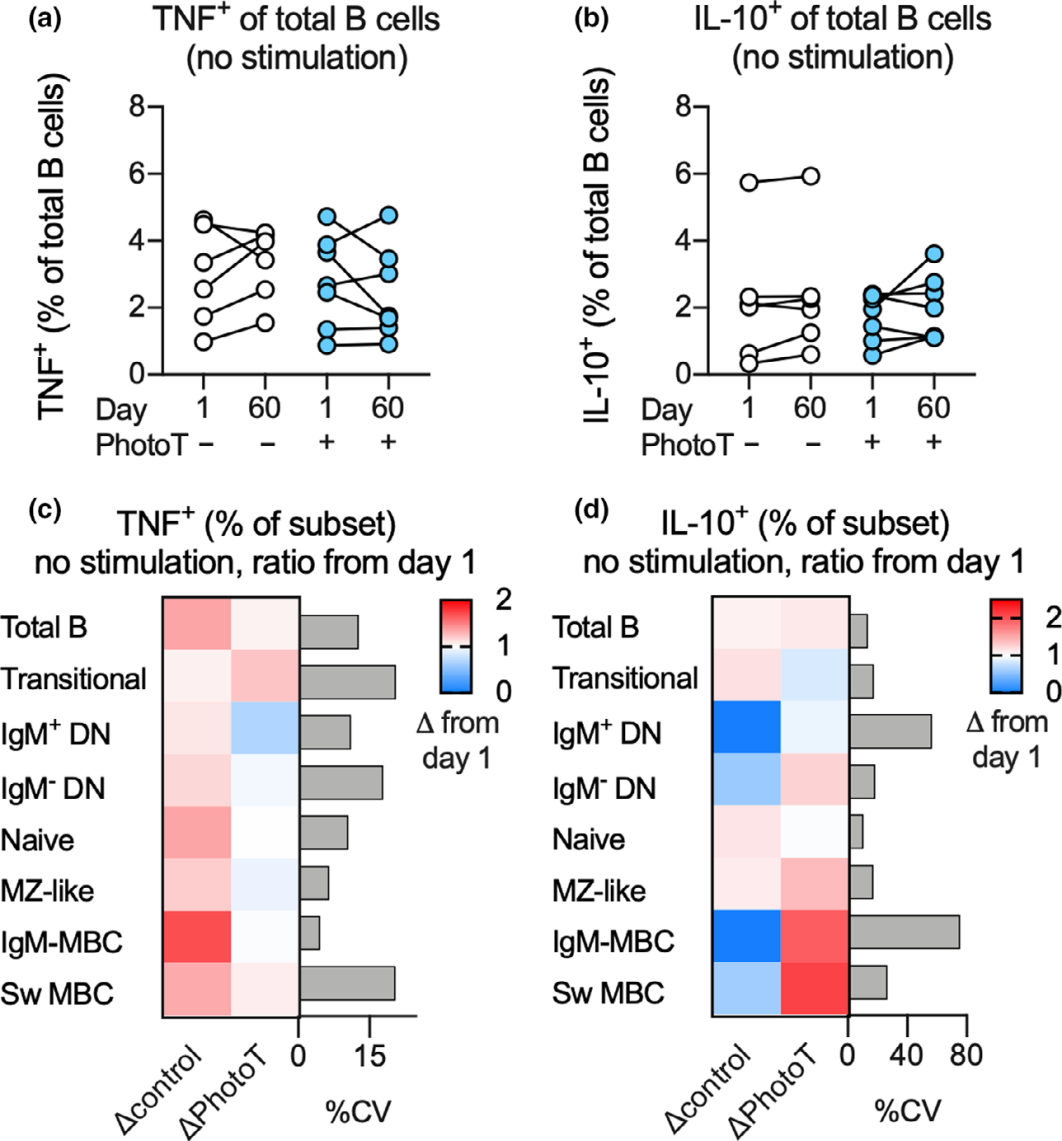
**(a–d)** Proportion of TNF^+^ **(a)** and IL‐10^+^ **(b)** cells within the total B‐cell population or change (Δ) in proportion of TNF^+^ **(c)** and IL‐10^+^ **(d)** cells, at day 60 compared to day 1 across each B‐cell subset in unstimulated cultures of PBMC from controls (−PhotoT) or participants administered phototherapy (+PhotoT). In **a, b**, data are displayed as individual data points for each participant. Data in **c, d** are displayed as mean change and coefficient of variation (CV) in each subset for *n*_control_ = 6, *n*_PhotoT_ = 7 individuals measured once. Significance of impact of phototherapy was calculated using multiple regression analysis for cytokine positive subset at day 60 adjusted for phototherapy and cytokine positive subset at day 1. No significant impact was observed. IgM‐MBC, IgM‐only MBC.

To elucidate whether phototherapy had an impact on B‐cell cytokine production *in vivo*, PBMCs isolated from participants prior to (day 1) and following phototherapy (day 60) were cultured, but not stimulated, and TNF and IL‐10 production was examined after 18 h. The results were visualised for total B cells at days 1 and 60 (Figure 3a and b) and using the ratio of TNF or IL‐10 production at day 60 compared to day 1 across all B‐cell subsets (Figure 3c and d). When the potential impact of phototherapy after 60 days was calculated using multiple regression adjusting for cytokine production at day 1 within the same individual, no impact of phototherapy on endogenous cytokine production was observed in these unstimulated cells (Figure 3a–d).

**Figure 4:**
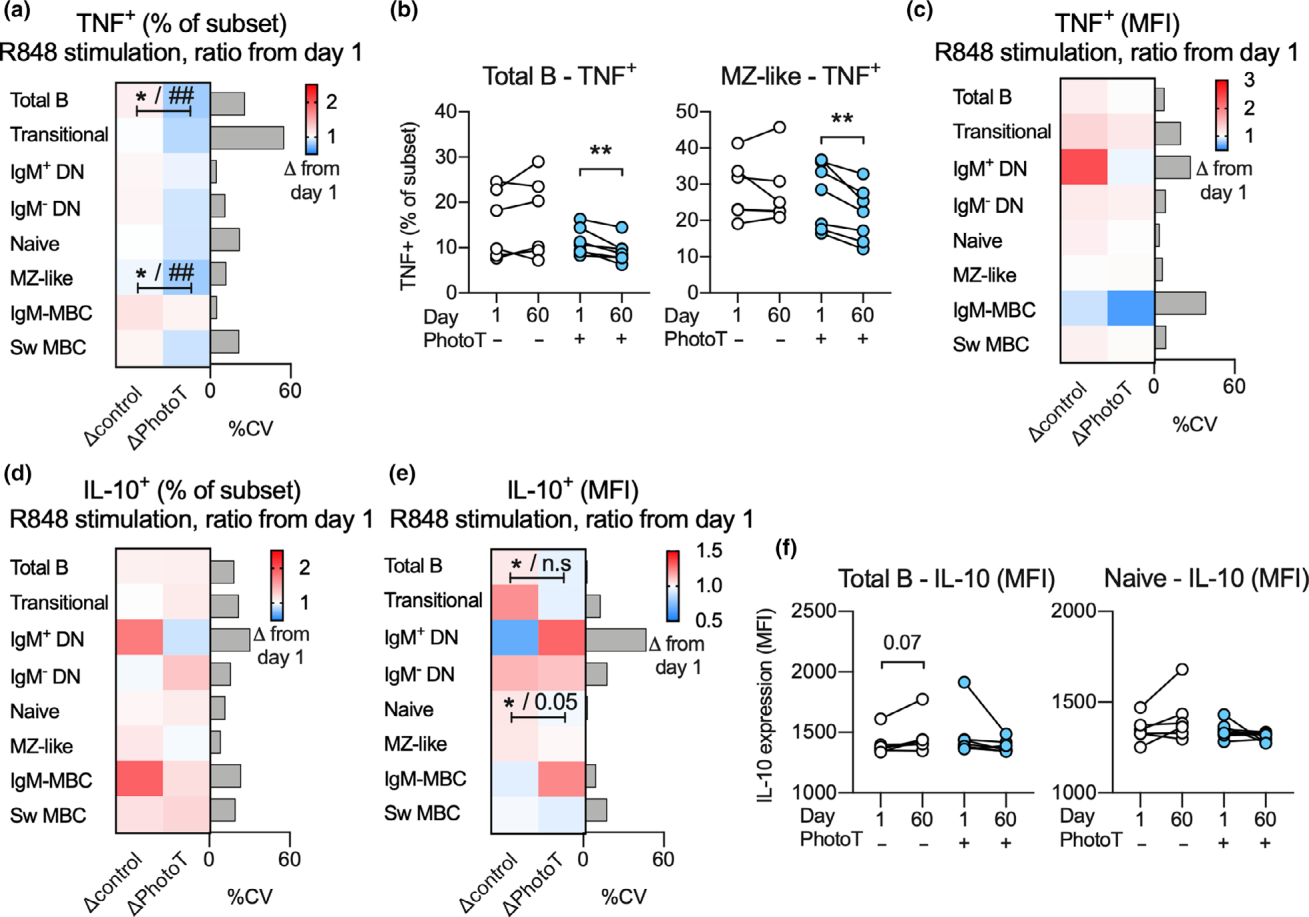
**(a, b)** Change (Δ) in proportion of TNF^+^ cells following R848 stimulation of PBMCs at day 60 compared to day 1 in controls (−PhotoT) and participants administered phototherapy (+PhotoT) across all B‐cell subsets **(a)** and specifically in total B cells and MZ‐like B cells **(b)**. **(c)** Change in expression of TNF (MFI) across all TNF^+^ subsets over the same period. **(d)** Change in proportion of IL‐10^+^ cells following R848 stimulation across all B‐cell subsets. **(e, f)** Change in expression of IL‐10 (MFI) across all IL‐10^+^ subsets **(e)** and specifically in IL‐10^+^ total B cells and IL‐10^+^ naive B cells **(f)**. Data are displayed as the mean change in proportion or expression (MFI) and coefficient of variation (CV) in each subset or as data points connected for each participant; *n*_control_ = 6, *n*_PhotoT_ = 7 measured once. Significance of impact of phototherapy was calculated using multiple regression analysis for cytokine positive subset (%) or cytokine level (MFI) at day 60 adjusted for phototherapy and cytokine positive subset (%) or cytokine levels (MFI) at day 1 and is indicated by *. For significant findings, a secondary analysis was employed to also adjust for the sex of each participant and is indicated by ^#^. Alternatively, a paired *t*‐test was used to detect changes in respective group. Level of significance is indicated as *P < 0.05, **^/##^ *P* < 0.01 or as a specified *P*‐value. IgM‐MBC, IgM‐only MBC; n.s., not significant.

After establishing that all B‐cell subsets were able to respond to R848 with TNF and IL‐10 production (Figure 2b and c), the impact of phototherapy on R848‐induced cytokine responses in stimulated B cells was assessed. To adjust for individual differences at day 1, as well as differences in sex between the groups, multiple regression analysis was used and results were visualised using the ratio of TNF or IL‐10 production at day 60 compared to day 1. In the total B‐cell population, as well as in the MZ‐like B‐cell subpopulation, the frequency of TNF^+^ cells was significantly lower in R848‐stimulated cells at day 60 in those who received phototherapy compared to controls. The significance of this observation was further strengthened when adjusting for sex in the secondary analysis (Figure 4a). These findings were also confirmed using paired analysis before and after phototherapy (Figure 4b). Further, we did not detect any impact of phototherapy on the quantity of TNF produced (as assessed by MFI) in TNF^+^ B‐cell subsets (Figure 4c). Phototherapy did not have any impact on the proportion of IL‐10^+^ B‐cell subsets (Figure 4d) although reduced IL‐10 production (as assessed by MFI) was observed in the IL‐10^+^ total B‐cell population as well as in naïve B cells in those who received phototherapy. In the secondary analysis, adjusting for sex, the impact of phototherapy was not significant between the two groups (Figure 4e). Further, the differences in IL‐10 production were not confirmed using paired analyses (Figure 4f). Paired analyses of non‐significant findings are displayed in Supplementary figure 2a–d.

**Figure 5:**
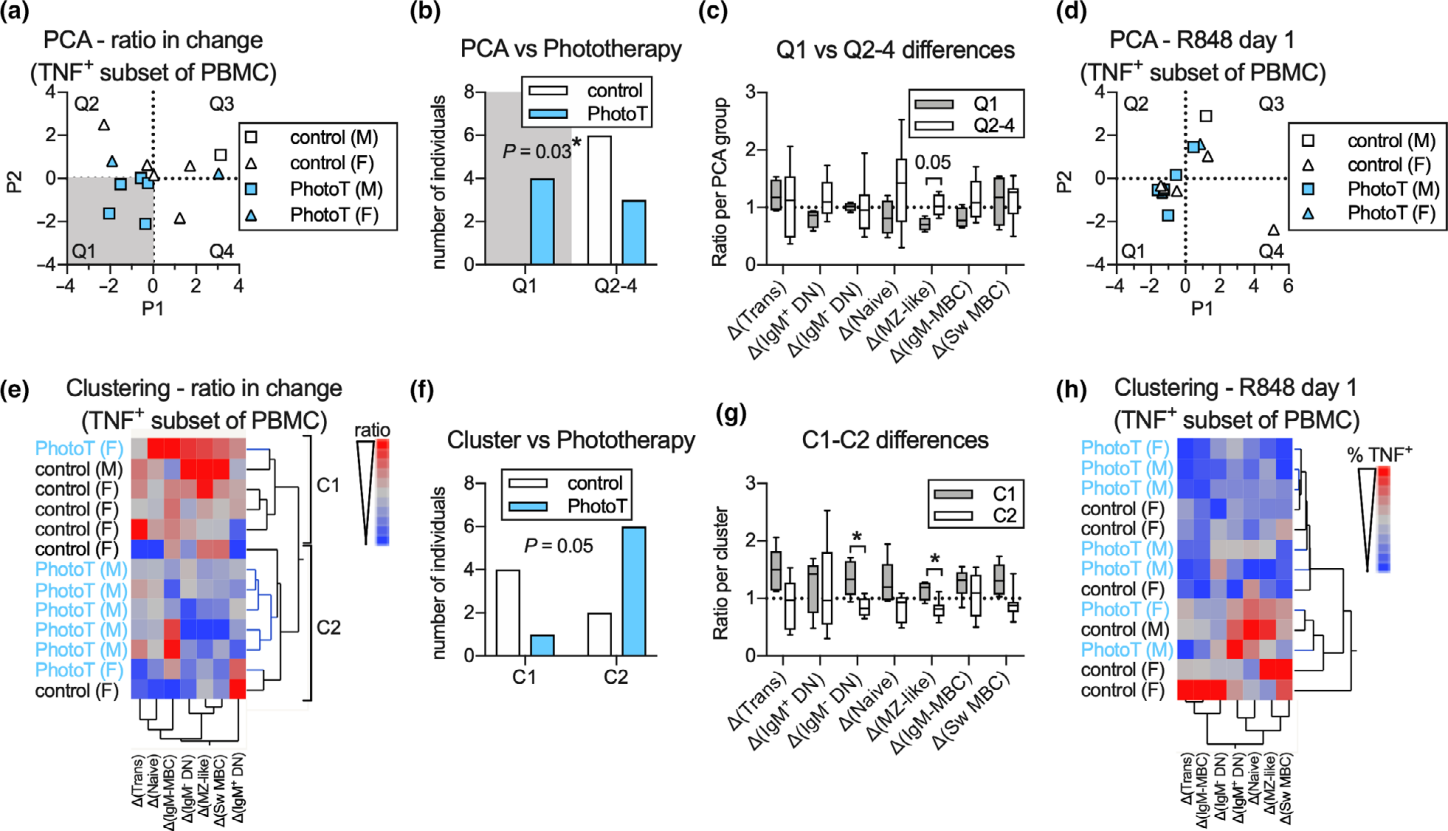
**(a–c)** Principal component analysis **(a)** of the ratios (Δ) of TNF^+^ cells as % of PBMC across all subsets in R848‐stimulated cells at day 60 compared to day 1 for controls and participants administered phototherapy, including distribution of participants in Q1 vs Q2‐4 **(b)** as well as contribution of changes in TNF^+^ B‐cell subsets between the two groups **(c)**. **(d)** Principal component analysis of all participants at day 1 prior to treatment. **(e–g)** Cluster analysis **(e)** and distribution of controls and participants administered PhotoT in cluster 1 (C1) and 2 (C2) **(f)** as well as mean ratios for each cell subset in C1 and C2 **(g)**. **(h)** Cluster analysis on all participants at day 1 prior to treatment. Data are displayed as individual data points, mean ratios or box/whiskers that display median and range for *n*_control_ = 6, *n*_PhotoT_ = 7 measured once. Proportional distribution in **b** and **f** was calculated using the chi‐square test, and differences in ratio for each subset were calculated using the Student’s *t*‐test, and level of significance is indicated as *P < 0.05 or as a specified *P*‐value. IgM‐MBC, IgM‐only MBC.

To assess whether subtle effects of phototherapy across all B‐cell subsets considered together could identify which individual belonged to which treatment group, we utilised principal component and cluster analyses on the ratio of TNF^+^ subsets (as % of PBMC) after stimulation at day 60 compared to day 1. Participants from the phototherapy group were more likely than controls to be located in quadrant 1 and display both negative P1 and P2 values (Figure 5a and b) and the difference between the two groups, following phototherapy treatment appeared to be driven by a decrease in TNF^+^ MZ‐like B cells over the 60‐day period (Figure 5c). As the two treatment groups differed in proportion of male and females, we assessed whether sex contributed to P1 or P2 prior to phototherapy and demonstrated that sex did not appear to contribute to either P1 or P2 (Figure 5d). In the cluster analysis, the majority of participants in the phototherapy group clustered together in cluster 2 (Figure 5e). This distribution was borderline significantly different from random distribution (*P* = 0.05; Figure 5f). The potential difference between clusters appeared driven by the change of TNF^+^ DN IgM^‐^ and TNF^+^ MZ‐like B‐cell subsets (Figure 5g). No pre‐existing clustering based on sex was observed (Figure 5h). This suggests that phototherapy induces changes in cytokine production by B cells stimulated via TLR7, which become more apparent when analysing all B‐cell subsets simultaneously. Using the same methodology, no separation of participants emerged in unstimulated samples (data not shown).

**Figure 6:**
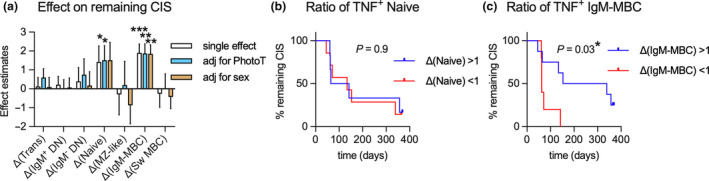
**(a)** Effect estimates of the ratios (Δ) of TNF^+^ B‐cell subsets as % of PBMC on time to MRI‐confirmed CIS to MS conversion using a parametric survival model as a single effect, adjusted for phototherapy (PhotoT) or sex. **(b, c)** Differences in time to CIS to MS conversion in participants with increasing or decreasing ratios of TNF^+^ Naive **(b)** or IgM‐only MBC (IgM‐MBC) **(c)** subsets during the 60‐day study period. In **a**, data are displayed as means with standard error for *n* = 13 measured once. In **a**, significance of effects was calculated using the Wald test. In **b, c,** significance of difference in time to relapse was calculated using the log‐rank test for *n*_ΔNaive> 1_ 6, = *n*_ΔNaive < 1_ = 7, *n*_ΔIgM‐MBC < 1_ = 5, *n*_ΔIgM‐MBC> 1_ = 8.

After establishing that phototherapy had a significant impact on how some B‐cell subsets respond to R848, we further investigated whether a change in cytokine responses in a particular B‐cell subset during the 60‐day study period was associated with longer time before MRI‐confirmed CIS to MS conversion. Using a parametric survival model, an increase in TNF^+^ naïve B cells and TNF^+^ IgM‐only MBC (as % of PBMC) over the study period was associated with a longer time before relapse (Figure 6a). No impact of phototherapy on this association was observed. By adjusting for sex, the association with changes in TNF^+^ naïve cells was rendered non‐significant. To test the use of these changes for predicting future relapses, we divided the trial participants into groups, those with increasing and those with decreasing TNF^+^ naïve or IgM‐only MBC frequencies between day 1 and day 60, and compared their time before conversion from CIS to MS (Figure 6b and c). Supporting the loss of significance after adjusting for sex, there was no difference in participants with increasing versus decreasing ratios of TNF^+^ naïve B cells following stimulation (Figure 6b). In contrast, participants with increasing TNF^+^ IgM‐only MBC displayed a significantly longer time before MS conversion compared to those with decreasing ratios of TNF^+^ IgM‐only MBC over the same period of time (Figure 6c). This exploratory analysis suggests that changes to innate responses and/or activation of IgM‐only MBC over time may be associated with conversion from CIS to MS.

## Discussion

A 60‐day course of narrowband UVB phototherapy has been examined as a novel strategy to reduce the risk of CIS to MS conversion. A significant impact of phototherapy on circulating B‐cell subsets was observed at 60 days in the phototherapy group, namely a reduction in the proportion of Sw MBC and MZ‐like B cells (each ~ 10–15% of total B cells), and an increase in the proportion of naïve B cells (~60% of total B cells). Compared with the control group (no phototherapy), in those participants receiving phototherapy, we also discovered a functional change at 60 days in circulating B cells, namely fewer cells producing TNF in response to TLR7 stimulation. The effect was significant in all B cells considered together, and upon B‐cell subset analysis, in the MZ‐like B cells. When responses by seven B‐cell subsets were analysed together using multivariate methods, the treatment groups clearly separated. The clarity of the results was surprising considering that PBMCs were available for analysis from only 13 participants of the PhoCIS trial, specifically 7 who received narrowband UVB phototherapy and 6 who were randomly assigned to the control group. Finally, quantitative changes from day 1 to day 60 in the ability of the participants’ IgM‐only MBC to produce TNF in response to R848 significantly distinguished those who converted to MS early in the 12 month trial versus those who relapsed late or not at all.

The beneficial clinical effects of anti‐CD20 therapy, which targets the majority of B‐cell subsets but not plasma cells, suggest that B cells may contribute to MS pathology through additional mechanisms to antibody production, such as antigen presentation or cytokine production following innate immune stimulation, as reviewed.^19^ However, not all biologics targeting B‐cell numbers and function have been successful,^22^ and thus, more subtle approaches are required that target only pathogenic subsets of B cells.

In this study, B‐cell subsets were interrogated for their responses to R848; a ligand for TLR7, a receptor that responds *in vivo* to RNA from either dying cells or single‐stranded RNA of viruses such as human immunodeficiency virus or influenza viruses.^18^ Stimulation with R848 increased the percentage of B‐cell subsets synthesising TNF and IL‐10, and stimulation was necessary to detect reduced numbers of TNF‐producing B cells from those participants administered phototherapy. To account for any inter‐individual variability, differences in sex and possible effects of diurnal variation on B‐cell function between the two treatment groups, responses to R848 stimulation at day 60 were adjusted using the same response at day 1 (before phototherapy was commenced) as well as sex. The highest proportions of R848‐induced TNF‐expressing cells were detected in IgM‐only MBC (CD27^+^/IgD^−^/IgM^+^) and Sw MBC (CD27^+^/IgD^−^/IgM^−^) with 20–45% responding (Figure 2b). However, a thorough investigation did not identify a significant effect of phototherapy on the percentage of these cells expressing TNF or the amount expressed per B‐cell subset. Instead, a significant effect of phototherapy was detected in MZ‐like B cells (CD27^+^/IgD^+^) whose numbers were also reduced at 60 days by phototherapy. It is notable that at 60 days there were increased frequencies of naive B cells in blood from those administered phototherapy;^14^ however, a low proportion of naive B cells produced TNF in response to R848,^23^ possibly due to low TLR7 expression compared to memory subsets.^24^ The proportion of naive B cells that responded to TLR7 stimulation with IL‐10 production was lower compared to the proportion that responded with TNF production (up to 6%), and significant effects of phototherapy at 60 days were not detected. This result for IL‐10 does not support the hypothesis that narrowband UVB phototherapy may induce greater numbers of potentially immune‐regulatory IL‐10‐producing B cells.^25^

The relevance of reduced TNF production by MZ‐like B cells after phototherapy requires further investigation. MZ‐like B cells represent a population of B cells that respond primarily to microbial signatures via TLRs and can undergo immunoglobulin class switching when activated with cytokines and TLR ligands.^26^ In particular, MZ‐like B cells may be activated by neutrophils and undergo class switching in response to interferon‐γ.^27^ They may also enter germinal centre reactions.^27^ Phototherapy may therefore dampen the activity of a B‐cell subpopulation that is critical to the development of early T‐cell‐independent B‐cell responses. Alternatively, MZ‐like B cells may be the unswitched memory B cells that induce CD4^+^ T‐cell autoproliferation in patients with MS.^28^ As such, a decline in MZ‐like B‐cell numbers and the frequency of TNF^+^ MZ‐like B cells following phototherapy could signal reduced formation of B cells with polyclonal reactivity (potentially against self) after phototherapy.

To compensate for the relatively few participants included in the study, we utilised multivariate analytical strategies to identify effects of phototherapy that were only observable when all cell subsets were considered collectively. We focused on the TNF responses at day 60 (adjusted for response at day 1) as phototherapy did not induce any significant effects on IL‐10 responses. Using principal component analysis, a subgroup of participants administered phototherapy could be distinctly identified. Differences in response by MZ‐like B cells appeared to be related to the quadrant a participant was assigned to. Using cluster analysis, participants receiving phototherapy and those not receiving phototherapy could be mostly separated, with significant changes observed in the MZ‐like B cells and IgM^‐^ DN cells (CD27^−^/IgD^−^). Further, the participants of the trial were all defined as CIS but, as it is an evolving condition, there is likely to have been heterogeneity amongst them, including their B‐cell profile and responses to innate signals.

Regardless of the phototherapy intervention, decreasing frequencies of TNF^+^ IgM‐only MBC following TLR7 stimulation between days 1 and 60 sampling points was predictive of participants that relapsed within the first 100 days of the trial. These cells, while only representing ~ 1–2% of B cells (Figure 1c), contained a high proportion of TNF‐producing cells following culture with R848. Like Sw MBC, IgM‐only MBC are antigen‐ and germinal centre‐experienced and can be a source of long‐lived immunity.^29^ However, they are phenotypically distinct from Sw MBC^26^ and with different functional responses including increased responses to endosomal dsDNA, antigen presentation^30^, ^31^ and their capacity to form plasmablasts independent of T‐cell help.^32^ Additionally, IgM‐only MBC can differentiate to plasma cells and secrete IgM, which one study found had higher affinity for viral antigens than class switched IgG antibodies of the same specificity.^33^ The role of these B cells in progression from CIS to MS warrants further investigation.

The signals generated in UVB‐irradiated skin that alter the number and function of circulating B cells are not clear. Although UVB can only reach to the upper dermis of human skin, many systemic UVR‐induced mediators have been proposed for systemic immunoregulation that can be measured three days after UVR exposure.^7^, ^8^ No evidence to date supports UVR‐induced 25(OH)D as being responsible. In the PhoCIS trial, significantly increased serum levels of 25(OH)D at days 60 and 90 were observed^11^ but trials of supplementation of MS patients with vitamin D have generally reported an impact on T‐cell populations and not B‐cell subsets as reported here.^34^, ^35^ However, changes to cells of the haematopoietic system in the bone marrow may be affected by narrowband UVB phototherapy. In murine models, chronic low‐dose broadband UVR mediated functional changes in dendritic cells derived from myeloid progenitors in bone marrow, including enhanced glycolytic flux and a reduced capacity to prime immune responses.^36^, ^37^, ^38^ An effect on B‐cell progenitors in bone marrow requires further research.

In summary, two effects of narrowband UVB phototherapy have been detected in B cells from participants in the PhoCIS trial. Here, we show that fewer total and MZ‐like B cells in the blood of participants who had received narrowband UVB phototherapy over the past 60 days responded to TLR7 stimulation with TNF production. This result complements our previous finding of reduced numbers of MBC and MZ‐like B cells in those participants. Both outcomes support a dampening effect of phototherapy on B cells, and cells that anti‐CD20 therapies have suggested are associated with MS progression. In addition, we propose that the B‐cell function curtailed by phototherapy is pathogenically important and may reflect priming effects of phototherapy that allow for reduced responses by B cells to polyclonal activation.

## Methods

### Participants and the PhoCIS trial

Thirteen of the 20 participants with CIS recruited for the PhoCIS trial had sufficient biobanked paired peripheral blood mononuclear cell (PBMC) samples (day 1 and day 60) for inclusion in this sub‐study; 7 were randomised to receive phototherapy; and 6 were controls and did not receive phototherapy. The study protocol has been reported; participants undergoing phototherapy received 24 sessions over a period of 2 months.^11^

### PBMC collection and culture

Peripheral blood mononuclear cells were isolated from peripheral venous blood and cryopreserved in 10% dimethyl sulfoxide (DMSO) in foetal bovine serum (FBS; HyClone; Cytiva, Marlborough, MA, USA). For the study, PBMCs were thawed in 10% FBS in RPMI 1640 medium, as previously reported.^14^, ^39^ For culture with R848 and intracellular cytokine detection, approximately 8 × 10^5^ cells were cultured in RPMI 1640 medium supplemented with 10% FBS, 5 µg mL^−1^ gentamicin, 2 mm L‐glutamine and 50 µm 2‐β‐mercaptoethanol (all Sigma‐Aldrich; Merck, North Ryde, Australia)^12^ and 1 µg mL^−1^ of GolgiPlug (BD Biosciences, North Ryde, Australia) as well as 500 ng mL^−1^ of R848 (InvivoGen, San Diego, CA, USA) as indicated for 18 h in U‐bottom shaped 96‐well polypropylene plates at 37°C in 5% CO_2_.

Approximately 10^6^ cells were stained for surface markers directly after cryopreservation and after culture. For staining after storage, reconstituted PBMCs were washed in phosphate‐buffered saline (PBS; Gibco; Thermo Fisher Scientific, Scoresby, Australia) and then incubated with live/dead stain according to the manufacturer’s recommendation (FVS575V; BD Biosciences) for 30 min at 4°C. Cells were washed with flow cytometry buffer (4% FBS in PBS) and then incubated with a cocktail of antibodies specific for surface markers. Monoclonal antibodies generated in mice against CD19 (BUV737 clone SJ25C1), CD20 (BUV737 clone 2H7), CD24 (BV786 clone ML5), CD27 (BB700 clone L128), CD38 (BV510 clone HIT2), CD64 (FcγRI; BV711 clone 10.1), IgM (BUV395 clone G20‐127) and IgD (PE‐CF594 clone IA6‐2) were obtained from BD Biosciences. Following incubation, cells were washed and fixed for 10 min at room temperature with 2% paraformaldehyde, then washed twice and resuspended in flow cytometry buffer and stored at 4°C until analysis.

Following culture, cells were washed with cold PBS and stained for viability, followed by surface markers, as above. After surface staining, a Cytofix/Cytoperm kit (BD Biosciences) was used to fix, permeabilise and wash cultured cells. For intracellular staining, antibodies against TNF (PE‐Cy7 clone MAb11) and IL‐10 (PE clone JES3‐19F1), both from BD Biosciences, were incubated with cells for 30 min at 4°C. Following incubation, cells were washed and thoroughly resuspended in flow cytometry buffer and stored at 4°C until analysis.

### Flow cytometry analysis

Flow cytometry data were acquired using the BD LSRFortessa (BD Bioscience). In order to ensure consistency between the median fluorescence intensity (MFI) values obtained for intracellular cytokines in separate experimental batches, ultra rainbow calibration beads (Spherotech, ProSciTech, Thuringowa, Australia) were used to adjust photomultiplier (PMT) voltages of fluorophore channels to target MFI values for bead peaks. Approximately 150 000 PBMCs were acquired for each sample, and data analysis and compensation were carried out using FlowJo software (v10.6.1, BD Biosciences).

Following exclusion of doublets and gating on cells with PBMC‐like FSC/SSC properties, viable cells were determined by negative staining for FVS575V (not shown). B cells were identified as CD64^−^/CD19^+^/CD20^+^. Subsequently, plasmablasts/antibody‐secreting cells (CD27^hi^/CD38^hi^) were excluded from the B‐cell population (not shown), and B cells were gated into transitional, naive, MZ‐like, DN and MBC. The MBCs were further divided into IgM‐only MBCs and Sw MBCs (IgM^−^) (Figure 1a). Similarly, DN B cells were subdivided into IgM^+^ and IgM^−^ subpopulations.

### Statistical analysis

The statistical significance of the impact on B‐cell subsets by phototherapy was investigated using standard least squares multiple regression model, as the primary analysis, to adjust for individual differences in baseline levels/ responses at day 1, prior to treatment. If a significant impact of phototherapy was observed, a secondary analysis was performed to also adjust for the difference in number of males/females in the two treatment groups. For continuous data, the statistical significance of differences between groups was assessed using the Student’s *t*‐test, paired or unpaired, as appropriate, for comparison of baseline (day 1) and day 60 levels within individuals. For categorical data, the statistical significance of difference between groups was assessed using Pearson’s chi‐square test. To assess the statistical significance of the impact of independent variables on time to MS conversion, a parametric survival model was used to screen changes in B‐cell subsets, and for comparison between groups, a log‐rank test was used. Hierarchical clustering was performed using the Ward method. All statistical analyses were performed using JMP 13.0 (SAS, Lane Cove, Australia) and visualised in Prism 8.02 (GraphPad Software, San Diego, CA).

## Author Contributions

**Stephanie Trend:** Conceptualization; Formal analysis; Investigation; Methodology; Project administration; Resources; Validation; Visualization; Writing‐original draft; Writing‐review & editing. **Jonatan Leffler:** Formal analysis; Methodology; Validation; Visualization; Writing‐original draft; Writing‐review & editing. **Matthew N Cooper:** Formal analysis; Validation; Writing‐review & editing. **Scott N Byrne:** Investigation; Visualization; Writing‐review & editing. **Allan G Kermode:** Funding acquisition; Resources; Writing‐review & editing. **Martyn French:** Conceptualization; Methodology; Visualization; Writing‐review & editing. **Prue H Hart:** Conceptualization; Formal analysis; Funding acquisition; Investigation; Methodology; Project administration; Resources; Supervision; Validation; Visualization; Writing‐original draft; Writing‐review & editing.

## CONFLICT OF INTEREST

The authors have no conflicts of interest.
