# Supplementary material for: Narrowband UVB phototherapy reduces TNF production by B‐cell subsets stimulated via TLR7 from individuals with early multiple sclerosis
Source: Clin Transl Immunology. 2020 Oct 15;9(10):e1197. doi: 10.1002/cti2.1197 (PMC7561518; doi:10.1002/cti2.1197)
Supplement: Supplementary file 1 [file CTI2-9-e1197-s001.pdf]

Narrowband UVB phototherapy reduces TNF production by B cell subsets stimulated via TLR7 from individuals with early multiple sclerosis

Stephanie Trend, Jonatan Leffler, Matthew N Cooper, Scott N Byrne, Allan G Kermode, Martyn A French, Prue H Hart

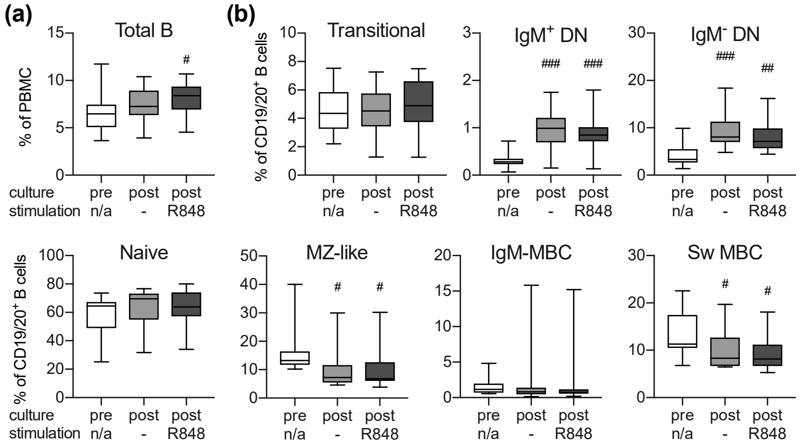

Supplementary Figure 1

(a-b) Abundance of total B cells (**a**) and proportion of B cell subsets within the B cell population (**b**) in PBMCs from all participants at day 1. Abundance was analysed pre-culture, and post 18 h culture without (-) and with R848 stimulation. Data are displayed as box/whiskers for median and range (n = 13) measured once. The significance of differences in abundance / proportion between *ex vivo* and cultures were calculated using Student's *t*-tests and indicated as; $^{\#}$, $P < 0.05$, $^{\#\#}$, $P < 0.01$, $^{\#\#\#}$, $P < 0.001$. IgM-MBC, IgM-only MBC.

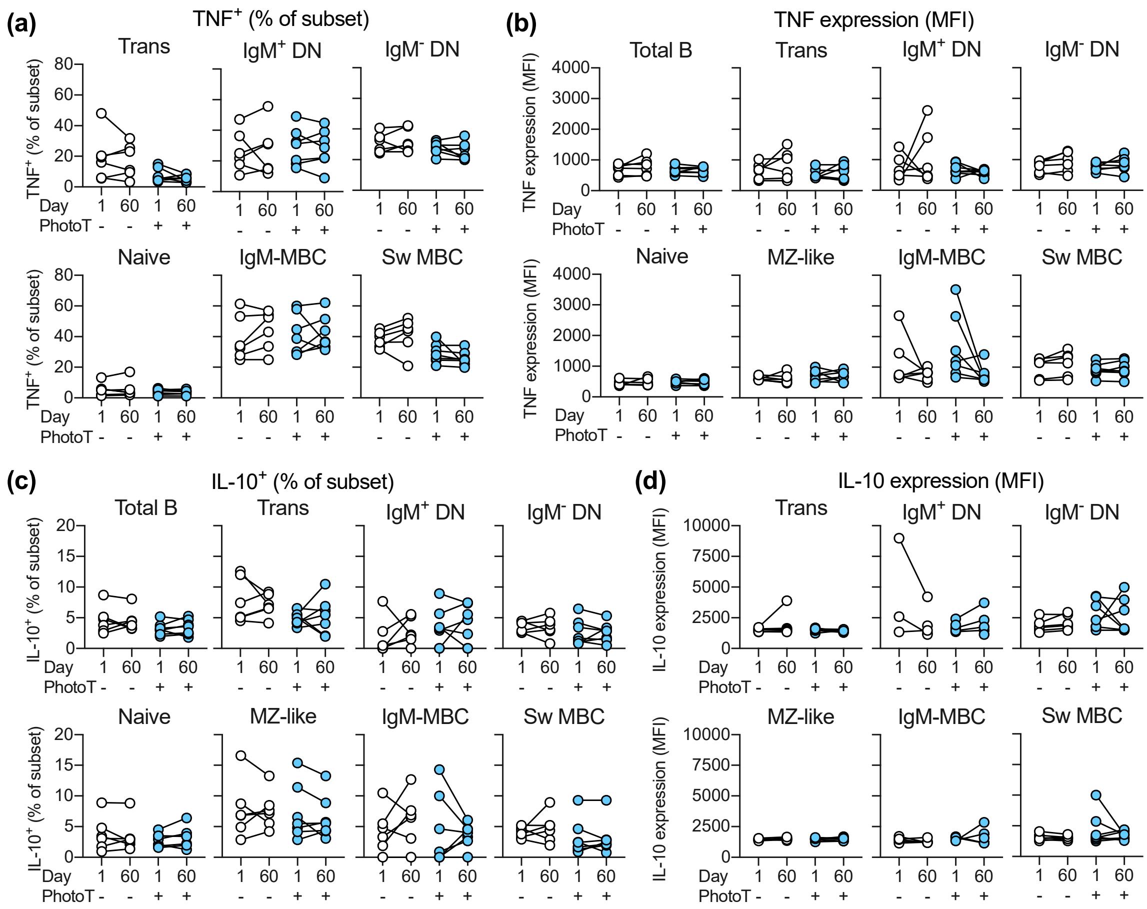

Supplementary Figure 2

(a-d) Change in proportion of TNF^{+} cells **(a)**, TNF expression (MFI) on TNF^{+} cells **(b)**, proportion of IL-10^{+} cells **(c)** and IL-10 expression (MFI) on IL-10^{+} cells **(d)** following R848-stimulation of PBMCs at day 60 compared to day 1 in controls (-PhotoT) and participants administered phototherapy (+PhotoT) across B cell subsets. Data are displayed as individual data points connected for each participant; $n_{control} = 6$, $n_{PhotoT} = 7$ measured once. Significance of impact of phototherapy was calculated using a paired *t*-test. No significant impact was observed. IgM-MBC, IgM-only MBC.
